# Development of microsatellite markers for the soft tick *Ornithodoros phacochoerus*

**DOI:** 10.1186/s13071-024-06382-7

**Published:** 2024-07-11

**Authors:** Florian Taraveau, David Bru, Carlos João Quembo, Hélène Jourdan-Pineau

**Affiliations:** 1 UMR ASTRE, CIRAD, INRAE, Campus de Baillarguet, 34398 Montpellier, France; 2 Central Region Office-Regional Veterinary Laboratory, Agricultural Research Institute of Mozambique, Chimoio, EN6.CP42 Mozambique

**Keywords:** Vector surveillance, Soft tick, *Ornithodoros*, Population genetics, Microsatellite markers, African swine fever

## Abstract

**Background:**

Soft ticks of the genus *Ornithodoros* are responsible for the maintenance and transmission of the African swine fever (ASF) virus in the sylvatic and domestic viral cycles in Southern Africa. They are also the main vectors of the *Borrelia* species causing relapsing fevers. Currently, no genetic markers are available for Afrotropical *Ornithodoros* ticks. As ASF spreads globally, such markers are needed to assess the role of ticks in the emergence of new outbreaks. The aim of this study is to design microsatellite markers that could be used for ticks of the *Ornithodoros moubata* complex, particularly *Ornithodoros phacochoerus*, to assess population structure and tick movements in ASF endemic areas.

**Methods:**

A total of 151 markers were designed using the *O. moubata* and *O. porcinus* genomes after elimination of repeated sequences in the genomes. All designed markers were tested on *O. phacochoerus* and *O. porcinus* DNA to select the best markers.

**Results:**

A total of 24 microsatellite markers were genotyped on two populations of *O. phacochoerus* and on individuals from four other *Ornithodoros* species. Nineteen markers were selected to be as robust as possible for population genetic studies on *O. phacochoerus*.

**Conclusions:**

The microsatellite markers developed here represent the first genetic tool to study nidicolous populations of *O. phacochoerus*.

**Graphical Abstract:**

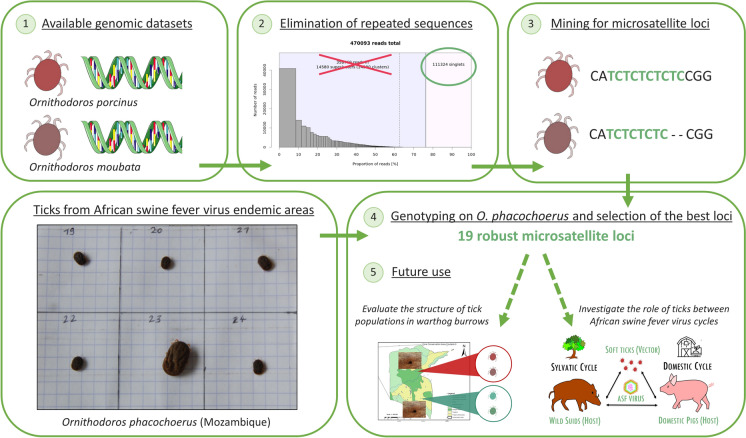

**Supplementary Information:**

The online version contains supplementary material available at 10.1186/s13071-024-06382-7.

## Background

*Ornithodoros phacochoerus* is an Afrotropical nidicolous soft tick widely distributed in Southern and Eastern Africa [1, 2]. *O. phacochoerus* belongs to a phylogenetically, ecologically, and biologically closely related complex of species, including *Ornithodoros moubata* and *Ornithodoros porcinus* [3]. The main hosts of these species include wild and domestic members of the *Suidae* family: warthog, bushpig, and domestic pig [4], but they can feed on any available host, including humans. There are two main health concerns with these species. First, they play a role as vectors in the transmission of the *Borrelia* responsible for relapsing fever, a neglected human disease [5]. Second, they are involved in the epidemiology of the African swine fever (ASF) virus by maintaining the virus in the wild and acting as a vector between *Suidae* hosts [6]. This latter role is particularly important to investigate at the interface between wild and domestic areas, as it is currently unknown to which extent *Ornithodoros* contribute to the transmission of ASF virus from warthogs to domestic pigs.

In this context, studying dispersal patterns of soft ticks is critical to assess their role in ASF virus transmission at the wild-domestic interface. Afrotropical *Ornithodoros* are nidicolous ticks that are mostly found in warthog burrows and in crevices from human facilities, including pig pens [1]. Population genetics represent an effective way to study the structure of these populations and to look for movement of ticks from burrows to other burrows or to nearby pig pens. Population genetics could also give insights into the mating behavior or the demography of soft ticks [7, 8]. Since no genetic markers were available for Afrotropical *Ornithodoros*, we decided to design microsatellite markers for *O. phacochoerus* and other closely related species using recently published genomic data of *O. porcinus* and *O. moubata* [9]. Microsatellite markers are cost-effective compared with more recent NGS-based methods for studies involving large number of samples and poor-quality or low quantities of DNA, as it is frequently the case for ticks and for ancient samples [10, 11]. Microsatellite markers were tested on two Mozambican populations of *O. phacochoerus* ticks and on four closely related *Ornithodoros* species. After analysis, 19 markers were selected for *O. phacochoerus* population genetic studies. The characteristics of these microsatellites are presented here.

## Methods

### Ticks

*O. phacochoerus* ticks were collected in Mozambique in 2021 and 2022, in the Coutada 9 Game Reserve from the district of Macossa (*n* = 29, late stage nymphs and adults, GPS coordinates: −17.7681, 33.8348) and in the Gorongosa National Park from the district of Gorongosa (*n* = 29, late stage nymphs and adults, GPS coordinates: −18.9775, 34.3521). They were shared under Material Transfer Agreement with the Mozambique Institute of Agricultural Research (IIAM) and in compliance with the Nagoya Protocol.

*O. moubata*, *O. porcinus*, *Ornithodoros maritimus*, and *Ornithodoros erraticus* ticks (*n* = 2 per species, late stage nymphs) came from colonies maintained at the CIRAD laboratory (Montpellier, France, member of the Vectopole Sud network) since 2008, 2012, 2015, and 2016, respectively.

The *O. moubata* colony originated from the Neuchâtel strain initially collected in Tanzania and maintained at the University of Neuchâtel (Switzerland). The *O. porcinus* ticks were initially sampled in Mahitsy (Madagascar) [12]. The *O. maritimus* ticks were collected in the field on the island of Carteau in Camargue (France) [13]. Finally, the *O. erraticus* ticks originated from the “Alentejo” strain collected in the field in Alentejo (Portugal) in 2013 and 2016 [14].

### DNA extractions

Nymph and adult ticks were washed in a 1% bleach bath for 30 s, then rinsed for 1 min in three consecutive baths of Milli-Q water to eliminate cuticular bacteria and avoid contamination for other downstream analyses such as ticks microbiota characterization [15]. Ticks were then cut and crushed individually using small scissors and pellet pestles. DNA was extracted from the crushed tick homogenate, using the standard protocol from the DNeasy^®^ Blood and Tissue genomic DNA extraction kit (Qiagen, Hilden, Germany). DNA extracts were finally eluted in 200 µl of elution buffer and stored at –20 ℃ until further use.

### Tick genomes and elimination of repeated sequences

After sequencing COI (primers: forward 5′-AATTTACAGTTTATCGCCT-3′, reverse 5′-CATACAATAAAGCCTAATA-3′ and forward 5′-GGAACAATATATTTAATTTTTGG-3′, reverse 5′-ATCTATCCCTACTGTAAATATATG-3′ [16]), the 12S rRNA gene (primers: forward 5′-AAACTAGGATTAGATACCCT -3′, reverse 5′-AATGAGAGCGACGGGCGATGT-3′ [17]), and the 16S rRNA gene (primers: forward 5′-CTGCTCAATGATTTTTTAAATTGCTGTGG-3′, reverse 5′- CCGGTCTGAACTCAGATCAAGT-3′ [18]), the ticks sampled in Mozambique were identified as *O. phacochoerus* (Additional file 1: Supplementary Dataset 1). Since no genome was available for this species, three genomic datasets from closely related species were used for microsatellite design [9]: one genome from *O. moubata* (cell line) and two genomes from *O. porcinus* (Kenya and Madagascar ticks). These genomic data were published by the Friedrich Loeffler Institute in Germany.

Tick genomes contain multiple sequence repeats, making microsatellite design a challenging task [19, 20]. To optimize this design, we employed the method published by Shah et al. for the elimination of repeated sequences in complex genomes [21]. For this purpose, the three tick genomes were screened for repeated sequences using RepeatExplorer2 clustering on Galaxy version 2.3.8.1 [22]. For each genome, reads identified as singletons by RepeatExplorer2 were retained for microsatellite mining, while sequences in clusters were discarded [21].

### Microsatellite design and selection

After elimination of repeated sequences, Palfinder [23] and Primer3 [24] from the Galaxy palfinder pipeline [25] were used to screen for microsatellite motifs and to design primer sequences for the potential markers. In total, 40,170 potential markers were obtained from the *O. moubata* genome, 18,689 from *O. porcinus* Kenya, and 33,006 from *O. porcinus* Madagascar. Sequences were then compared between the three genomes to keep only the potential markers that were common between at least two of the genomes. To be selected for further analyses, the markers also needed to be polymorphic between the two genomes or have a microsatellite pattern repeated at least eight times. In the end, 74 markers were kept from the comparisons between *O. moubata* and *O. porcinus* genomes, and 77 between *O. porcinus* Kenya and *O. porcinus* Madagascar genomes, for a total of 151 potential microsatellite markers (named from ms-1 to ms-151).

### PCR test on O. *phacochoerus* and genotyping

All 151 potential markers were amplified by touchdown polymerase chain reaction (PCR) using a 5′-end M13 extension (5′-CACGACGTTGTAAAACGAC-3′) on the forward primer and fluorescent M13 dye (FAM, VIC or NED) added to the PCR mix [26]. These first tests were performed on a batch of thirty samples from 15 different populations (two ticks per population) of *O. phacochoerus* from both Coutada 9 Game reserve and Gorongosa National Park, and two samples of *O. porcinus* as positive controls. The amplification mix consisted in 2 μL of DNA template, 10 μL 2 × DreamTaq Hot Start PCR Mastermix (Thermo Scientific, Courtaboeuf, France), 0.32 μL of M13 forward primer (0.16 μM), 0.4 μL of reverse primer (0.2 μM), and 0.4 μL of M13 dye (0.2 μM) in a final volume of 20 μL. The touchdown PCR program was set as follow: 98 ℃ for 3 min, then 10 cycles of 98 ℃ for 20 s, 60 − 0.5 ℃/cycle, for 30 s and 72 ℃ for 1 min, then 30 cycles of 98 ℃ for 20 s, 55 ℃ for 30 s, and 72 ℃ for 1 min, followed by a final extension step at 72 ℃ for 7 min.

Genotyping was performed at the GPTR laboratory (Great Regional Technical Platform of genotyping, AGAP Institut/CIRAD, Montpellier, France) with an ABI 3500xL Genetic Analyzer (Applied Biosystems, Foster City, CA). Of the 151 markers tested, 24 were selected according to the following criteria: successful amplification for *O. phacochoerus*, polymorphic between at least two *O. phacochoerus* samples, PCR products size ranging from 60 to 500 bp (for full sequences, see Additional file 2: Supplementary Dataset 2).

For the 24 selected markers, fluorescent-labeled forward primers (FAM, VIC, NED, or PET) were designed. Touchdown PCRs were performed in six multiplexes of four markers each. PCRs and genotyping tests were performed to adjust the concentration of each primer in the multiplexes (Additional file  3: Supplementary Table 1).

The amplification mix consisted in 2 μL of DNA template, 10 μL of 2× Type-it Microsatellite PCR Kit (Qiagen, Courtaboeuf, France), adjusted for volume of fluorescence-labeled forward primer and reverse primer for each of the four markers amplified in the PCR (according to the concentration chosen in Additional file  3) in a final volume of 20 μL. The touchdown PCR program was set as follow: 95 ℃ for 3 min, then 10 cycles of 95 ℃ for 20 s, 60 − 0.5 ℃/cycle, for 30 s and 72 ℃ for 1 min, then 30 cycles of 95 ℃ for 20 s, 55 ℃ for 30 s, and 72 ℃ for 1 min, followed by a final extension step at 72 ℃ for 7 min.

Formamide for denaturation and GeneScan-600 (LIZ) Size Standard Kit for ladder (Applied Biosystems, Foster City, CA) were added to the PCR products before genotyping by capillary electrophoresis at the GPTR platform.

The 24 selected markers were tested on two populations of *O. phacochoerus* ticks (Coutada 9 and Gorongosa) and on closely related (*O. porcinus* and *O. moubata*) and more distant (*O. maritimus* and *O. erraticus*) *Ornithodoros* species with two individuals for each species. For each marker, Sanger sequencing (Genewiz, Azenta Life Sciences, Leipzig, Germany) was performed on *O. phacochoerus* (Coutada 9), and a reference sequence was submitted to GenBank (Table 1).

**Table 1:** Characteristics of twenty-four microsatellite markers amplified in *O. phacochoerus*

Locus name	Primer sequences (5′–3′)	(Repeat motif)_n_	Null alleles	Stuttering	GenBank accession number
Subset of markers selected for *O. phacochoerus* (19 markers)					
ms-2	Fw: GTCGACAATTTCTCTCGCCC	(AC)_12_	No	No	PP813852
	Rv: TTCCCAAACAATGGGTCTCC				
ms-24	Fw: TGTTTACGACGGCATGAAGC	(GT)_8_	No	No	PP740832
	Rv: GCGGAAAATACGAAAGCTCG				
ms-30	Fw: AGGGTGCCCTCAATACAACG	(TG)_6_	No	No	PP740833
	Rv: TGTGTGCGCATGATGTAAGC				
ms-35	Fw: CTCAGGTGTCACCAGCAAGC	(AT)_9_	No	No	PP813853
	Rv: CCCGACAATGTCTAGGCTCC				
ms-48	Fw: TCTGCTTTTCAAGGCTGTGC	(AC)_7_	No	No	PP740831
	Rv: TTCGGAGCCTGTTACCTTGC				
ms-59	Fw: ATAGAGGCAAGATGGCAGGC	(TG)_16_	No	No	PP740844
	Rv: CCAGCTGTGCAAGTTCAAGG				
ms-61	Fw: CAGCGAAACAAGCAATGAGC	(ATT)_7_	Yes (Gor; *Cout9*)	*Yes (Cout9)*	PP740845
	Rv: AGCAAATCCCGGTTACAACG				
ms-63	Fw: CATGCTCACAGTGCTTGACG	(GT)_6_	No	No	PP740835
	Rv: TTGTCACATGACCAGAGGGG				
ms-71	Fw: TTCAGATTCACAACAGGGCG	(GAT)_5_	No	No	PP740837
	Rv: GCATTCAACGTGCTCTCACC				
ms-73	Fw: TTCGGATTCGAACAAACACG	(GA)_6_	No	No	PP813854
	Rv: GTTCGTGCCCTCTCACTTCC				
ms-76	Fw: TCTTACGCTGAACATTGGCG	(AG)_11_	Yes (Gor; Cout9)	No	PP740838
	Rv: AATTGCTACTGCACTGGGAGG				
ms-78	Fw: CTATCACGACGCCTCCTTCC	(GT)_3_	*Yes (Cout9)*	*Yes (Cout9)*	PP813855
	Rv: CTGAAGCTCAGCAATGACGG				
ms-81	Fw: CCCTTTGACAAACCGTAGGC	(TC)_6_	No	No	PP813856
	Rv: AAATCATTTTCGCCAGACCG				
ms-87	Fw: ATGAAGCGATCGTCCTACGG	(TC)_6_	No	No	PP740839
	Rv: GAGACGCTTTCCTGATTCGC				
ms-90	Fw: TGAATAACGGGGTAAAGCCG	(AG)_12_	No	No	PP740840
	Rv: TGGGAGTGCTGTATTCGTGC				
ms-101	Fw: GGCTCACGAAAATACCTCGC	(AG)_7_	No	No	PP740841
	Rv: CCAGCTAACGGTATGCTCCC				
ms-102	Fw: TGCGCCTACTGTGTACCACC	(TC)_7_	No	No	PP740842
	Rv: CCCGCAAGCTTCAGATAACC				
ms-111	Fw: CCAAAACACTGGATGAAGCC	(GGA)_6_	Yes (Cout9)	Yes (Cout9)	PP740847
	Rv: GTCGCTCAACCGTAGGAACC				
ms-117	Fw: CGCACTCATTGAGAGTTCGC	(TG)_5_	No	No	PP813858
	Rv: TTTAACGTTTCCGTGATGGC				
Discarded markers (5 markers)					
ms-46	Fw: TAGCGTGAACATAGCGGTGG	(AC)_6_	No	No	PP740834
	Rv: GGAGAAGTTTTCCCGGAAGG				
ms-64	Fw: CGGACAGAAATAGCGGAACC	(AT)_14_	No	No	PP740846
	Rv: ATAACCAAACGCAGGGATGC				
ms-66	Fw: CTTCCTTCTGATTGAGCGGC	(AAG)_5_	No	No	PP740836
	Rv: TTGAAGACACAAACGGTGGC				
ms-82	Fw: CAGTTCAGTTTACGCTCGGC	(AT)_5_	Yes (Cout9)	No	PP813857
	Rv: ACTCCATGAATTGGGTTCGG				
ms-96	Fw: CCACCCCTCTAGAACCCTCC	(TG)_6_	No	No	PP740843
	Rv: ATCTAAGCTGGCTGAACGGC				

For each locus are indicated: forward (Fw) and reverse (Rv) primers; the repeat motifs detected in the two genomes used for marker design; the presence (Yes) or absence (No) of null alleles and stuttering in Coutada 9 (Cout9) population or Gorongosa (Gor) population according to the analysis of blanks using Micro-Checker. Stuttering and null alleles are indicated in italics if correction was possible by pooling two ambiguous alleles

### Allelic diversity and statistics

Genotypes were read using GeneMapper^®^ v.6 software (Applied Biosystems, Waltham, MA). Allele bins were set manually after a review of all samples. Allele scoring was performed automatically according to the bin set designed for the marker, then manually checked by two different experimenters. Alleles were named according to their length in base pairs. When peaks were of low intensity in some of the samples, a threshold of peak intensity was set at 100 fluorescence units, below which the samples were not scored.

The resulting dataset was converted to Fstat and Micro-Checker format using CREATE [27]. Linkage disequilibrium p-values were calculated using Fstat v 2.9.4. [28], then corrected with a Benjamini and Yekutieli correction [29] on R version 4.2.3 (15 March 2023) [30]. Presence of null alleles, stuttering, and short allele dominance were tested using Micro-Checker [31]. When possible, correction for stuttering was performed by pooling alleles with overlapping signals, then stuttering was re-evaluated [32]. Observed and expected heterozygosity, Fis and Fst were calculated using Fstat v 2.9.4. [28].

After genotyping, two loci were readily eliminated: ms-46 which was monomorphic for all *O. phacochoerus* samples and ms-66 for which several individuals presented more than two alleles suggesting that the marker was duplicated in *O. phacochoerus* genome. Moreover, loci ms-96 and ms-64 were difficult to read due to the low quality of the profiles (low intensity profiles in which peaks were difficult to distinguish from each other). Finally, locus ms-82 was the only locus with an absence of heterozygote profiles in all samples leading to its elimination. The results obtained from these five markers are presented but they were not selected in the proposed subset of markers, leading to a subset of 19 markers selected out of 24 markers tested (Table 1). Sex-linkage of the loci could not be evaluated for the markers as information about the sex of each tick was not included in the dataset during DNA extraction steps.

## Results and discussion

The initial screening performed on *O. moubata* and *O. porcinus* genomic datasets led us to select 19 markers suitable for *O. phacochoerus* out of 24 markers tested (Table 1). Most of the loci selected presented dinucleotide repeats, with four loci having trinucleotide repeats. Several loci presented point mutations outside of the repeated dinucleotide or trinucleotide motifs, resulting in alleles with 1 nucleotide difference (in ms-48, ms-59, ms-73, ms-76, ms-81, ms-82, ms-90, ms-96, and ms-117). Such point mutations can increase the risk of stuttering [32] and forbid the calculation of evolutive distance between alleles according to the stepwise mutation model (SMM) which is based on the idea that microsatellite evolution happens by progressively adding or removing single repeat units [33].

The 19 selected markers were tested for linkage disequilibrium. Only loci ms-87 and ms-102 were suspected to be linked (genotypic disequilibrium test, permutations: 10,000, Benjamini and Yekutieli corrected *P*-value: 0.0978), while no sign of linkage disequilibrium was detected between any other pair of loci (genotypic disequilibrium test, permutations: 10,000, Benjamini and Yekutieli corrected *p*-value of 1.00). For future use of the markers presented here, we suggest to use locus ms-102 and to discard locus ms-87 to avoid having two markers potentially linked. Testing for stuttering revealed that three loci presented signs of stuttering in Coutada 9 population (ms-61, ms-78, and ms-111) and none in Gorongosa. To correct for stuttering, ambiguous alleles 313 and 314 were pooled together in locus ms-61, and ambiguous alleles 324 and 326 were pooled together in locus ms-78. This was sufficient to correct for stuttering. For locus ms-111, no ambiguous alleles were detected on the electropherograms, and no correction could be performed for this locus. Testing for null alleles revealed that out of 19 loci, 3 locus presented null alleles due to stuttering in Coutada 9 population (ms-61, ms-78, and ms-111), 1 in Gorongosa population independently from stuttering (ms-61), and 1 in both populations independently from stuttering (ms-76). Finally, no evidence of large allele dropout was detected in the 19 markers.

Allelic diversity ranged from two to six alleles per locus with variations within each population (Table 2). Wright’s fixation index F_IS_ was calculated in each of the two populations and for all samples. The absence of heterozygotes for locus ms-82 in all samples led to its elimination. Deviation from Hardy–Weinberg equilibrium was detected in four of the markers selected: ms-61, ms-76, ms-101, and ms-111. Loci ms-61 and ms-76 had deficit in heterozygotes. This was not surprising, since these loci showed signs of null alleles. Besides, positive F_IS_ can be due to consanguine mating, an expected feature in a nidicolous species such as *O. phacochoerus* [34]. Locus ms-111 presented a F_IS_ = 1 in Coutada 9 due to the absence of heterozygotes for this locus in this population. This is an unusual feature for a microsatellite marker, and this marker should be tested in more populations. Genotyping was performed on two populations of *O. phacochoerus* (Coutada 9 Game Reserve and Gorongosa National Park) separated by 150 km. Interestingly, warthogs (*Phacochoerus africanus*), which are the main host for *O. phacochoerus*, tend to remain in areas of less than 4 km^2^, and there is no warthog movement reported outside of the two conservation areas [35] [C. Quembo personal communication]. Consequently, it is very likely that there is no gene flow between these two areas which could explain some of the high values observed for Wright’s fixation index F_ST_ between the two populations (Table 2). However, some loci (especially loci ms-61, ms-87, and ms-111) presented low values of F_ST_ compared with the evaluation of F_ST_ over all loci. This could be the result of highly conserved loci or other issues previously mentioned (especially null alleles). In any case, the usefulness of these specific loci should be reevaluated for each dataset, as they might end up being irrelevant to screen for genetic differences in close located areas.

**Table 2:** Characterization of twenty-four microsatellite markers in *O. phacochoerus*

Locus name	All populations					Coutada 9		Gorongosa	
	*N*_alleles_	*H*_o_	*H*_e_	*F*_IS_	*F*_ST_	*N*_alleles_	*F*_IS_	*N*_alleles_	*F*_IS_
Subset of markers selected for *O. phacochoerus* (19 markers)									
ms-2	6	0.64	0.71	0.098	0.10	3	0.12	5	0.079
ms-24	2	0.29	0.25	−0.18	0.27	2	−0.18	1	NA
ms-30	2	0.24	0.21	−0.13	0.57	2	−0.077	2	−0.17
ms-35	4	0.54	0.55	0.022	0.12	2	−0.27	4	0.16
ms-48	6	0.40	0.34	−0.16	0.49	2	0.0	4	−0.17
ms-59	4	0.14	0.16	0.14	0.69	2	0.27	3	−0.042
ms-61	3	0.12	0.26	0.54	0.079	2	**0.49**	2	**0.56**
ms-63	3	0.21	0.20	−0.077	0.11	2	−0.084	2	0.0
ms-71	2	0.26	0.26	0.021	0.22	2	0.022	2	0.0
ms-73	4	0.40	0.46	0.13	0.35	2	0.036	4	0.18
ms-76	5	0.35	0.60	0.42	0.20	3	**0.40**	3	**0.42**
ms-78	6	0.48	0.55	0.12	0.23	3	0.24	5	0.057
ms-81	4	0.53	0.42	−0.24	0.41	2	−0.26	2	−0.23
ms-87	3	0.32	0.32	0.0040	0.080	2	0.036	3	−0.067
ms-90	6	0.12	0.12	−0.028	0.79	3	−0.011	4	−0.038
ms-101	3	0.50	0.41	−0.21	0.38	2	−0.018	2	−**0.40**
ms-102	4	0.45	0.49	0.089	0.22	2	0.14	3	0.042
ms-111	2	0.069	0.27	0.74	0.0051	2	**1.0**	2	0.27
ms-117	6	0.72	0.69	−0.050	0.11	3	−0.055	4	−0.046
All loci		0.36	0.38	0.069	0.30		**0.087**		0.053
Discarded markers (5 markers)									
ms-46	1	0.0	0.0	NA	0.0	1	NA	1	NA
ms-64	3	0.29	0.25	−0.18	0.60	1	NA	2	−0.18
ms-66	4	NA	NA	NA	NA	2	−0.20	4	NA
ms-82	2	0.0	0.25	1.0	0.23	2	**1.0**	1	NA
ms-96	2	0.28	0.26	−0.059	0.45	2	−0.038	2	−0.064

For each locus, for all samples are indicated (from both populations): number of alleles (*N*_alleles_), observed heterozygosity (*H*_o_), expected heterozygosity (*H*_e_), *F*_IS_, and *F*_ST_ between the two populations. For each locus, in each population are indicated: number of alleles (*N*_alleles_) and *F*_IS_ (text in bold if the value is significantly different from zero after 10,000 randomizations)

All 24 microsatellite markers were tested in four other *Ornithodoros* species. *O. porcinus* and *O. moubata*, which belong to same complex of species as *O. phacochoerus*, presented successful amplification for respectively 23 and 13 markers. In contrast, more distant species *O. maritimus* and *O. erraticus* showed successful amplification for four and zero loci respectively. This suggests that the markers developed here are quite specific to Afrotropical *Ornithodoros* species (Table 3).

**Table 3:** Genotyping success and allele size range of twenty-four microsatellite markers in five *Ornithodoros* species

Locus name	*O. phacochoerus*	*O. porcinus*	*O. moubata*	*O. maritimus*	*O. erraticus*
ms-2	+(303–325)	+(286)	+(289–291)	+(311)	–
ms-24	+(305–307)	+(293)	–	–	–
ms-30	+(196–212)	+(222)	+(224)	–	–
ms-35	+(67–75)	+(74)	+(60–62)	–	–
ms-46	+(383)	+(377)	+(389)	–	–
ms-48	+(259–272)	+(242)	+(281)	–	–
ms-59	+(422–435)	+(409)	+(411–425)	+(374)	–
ms-61	+(308–316)	+(310–312)	+(239–308)	–	–
ms-63	+(404–408)	+(410)	–	–	–
ms-64	+(463–477)	+(470)	+(480)	–	–
ms-66	+(356–391)	+(365–368)	–	–	–
ms-71	+(204–210)	+(210)	+(210)	–	–
ms-73	+(416–421)	+(454)	–	–	–
ms-76	+(383–390)	+(205)	–	–	–
ms-78	+(304–344)	+(293)	+(298–302)	–	–
ms-81	+(446–462)	+(446)	+(444)	–	–
ms-82	+(473–478)	–	–	–	–
ms-87	+(445–513)	+(475)	–	–	–
ms-90	+(446–464)	+(450)	–	+(452)	–
ms-96	+(336–337)	+(341)	+(336)	–	–
ms-101	+(148–156)	+(156–158)	+(143–152)	–	–
ms-102	+(267–279)	+(269)	–	–	–
ms-111	+(109–112)	+(121)	–	–	–
ms-117	+(447–458)	+(436)	–	+(456)	–

Two individuals were used for each species studied (except *O. phacochoerus* with 54 individuals). Successful amplification and allele detection is indicated as “ + ” followed by the range of allele size detected in brackets, unsuccessful amplification or allele detection is indicated as “ – ”

## Conclusions

We present here 24 microsatellite markers designed for the Afrotropical soft tick *O. phacochoerus*. We selected a subset of 19 markers that were relevant in this species. Design methodology and protocols for amplification of the markers were provided here. These markers will compensate for the lack of genetic tools available for Afrotropical *Ornithodoros* and help to investigate the role of this tick vector in ASF epidemiology. These markers will also help to understand the structure of populations and breeding patterns in nidicolous soft tick species and their dispersal abilities between warthog burrows.

### Supplementary Information

Additional file 1: Dataset 1. Sequencing of *Ornithodoros* ticks collected in Mozambique for species identification for COI, 12S rRNA gene, and 16S rRNA gene. Sequences assembled using Geneious.

Additional file 2: Dataset 2. Sequences of the twenty-four microsatellite markers designed.

Additional file 3: Table S1. Repartition of twenty-four microsatellite markers in three multiplexes for amplification and genotyping on *O. phacochoerus* samples. For each genotyping multiplex (named Plex1 to Plex3), two PCR were performed separately (PCR1 and PCR2) with four loci (Column Locus name) in each PCR marked with four different fluorochrome (Column Fluorescent dye). According to preliminary tests, PCR products were diluted (1:dilution) before being pooled together to form the genotyping multiplex. For each locus, the expected sequence length based on *O. porcinus* genomic data is indicated as it was taken in consideration for the repartition of fluorescent dyes between the loci (two loci with the same dye in the same genotyping multiplex needed to have different lengths to be distinguished in the final electropherogram). Finally, all fluorescent dyes did not lead to the same levels of amplification during PCR, consequently, primer concentrations were adjusted for each locus (Primer concentration) in each PCR to obtain similar level of fluorescence in the end. ^1^ The sequence size for locus ms-76 in *O. phacochoerus* ended up being longer (386bp) than the expected size.

## Data Availability

The datasets supporting the conclusions of this article are included within the article (microsatellite primers), its supplementary information (microsatellite sequences), and on the online Dryad repository, 10.5061/dryad.h44j0zpt3 (genotyping dataset).

## Abbreviations

ASF: African swine fever
PCR: Polymerase chain reaction

## References

[1] Vial L (2009). Biological and ecological characteristics of soft ticks (*Ixodida*: *Argasidae*) and their impact for predicting tick and associated disease distribution. Parasite Paris Fr.

[2] Leeson HS (1952). The recorded distribution of *Ornithodoros moubata* (*Murray*) (*Acarina*). Bull Entomol Res.

[3] Bakkes DK, De Klerk D, Latif AA, Mans BJ (2018). Integrative taxonomy of Afrotropical *Ornithodoros* (*Ornithodoros*) (*Acari*: *Ixodida*: *Argasidae*). Ticks Tick-Borne Dis.

[4] Peirce MA (1974). Distribution and ecology of *Ornithodoros moubata porcinus Walton* (*Acarina*) in animal burrows in East Africa. Bull Entomol Res.

[5] Cutler SJ, Abdissa A, Trape J-F (2009). New concepts for the old challenge of African relapsing fever borreliosis. Clin Microbiol Infect Off Publ Eur Soc Clin Microbiol Infect Dis.

[6] Jori F, Bastos A, Boinas F, Van Heerden J, Heath L, Jourdan-Pineau H (2023). An updated review of *Ornithodoros* ticks as reservoirs of African swine fever in sub-Saharan Africa and Madagascar. Pathog Basel Switz.

[7] Chevillon C, Koffi BB, Barré N, Durand P, Arnathau C, de Meeûs T (2007). Direct and indirect inferences on parasite mating and gene transmission patterns: pangamy in the cattle tick *Rhipicephalus* (*Boophilus*) *microplus*. Infect Genet Evol.

[8] Huber K, Jacquet S, Rivallan R, Adakal H, Vachiery N, Risterucci AM (2019). Low effective population sizes in *Amblyomma variegatum*, the tropical bont tick. Ticks Tick-Borne Dis.

[9] Forth JH, Forth LF, Lycett S, Bell-Sakyi L, Keil GM, Blome S (2020). Identification of African swine fever virus-like elements in the soft tick genome provides insights into the virus’ evolution. BMC Biol.

[10] Hauser SS, Athrey G, Leberg PL (2021). Waste not, want not: microsatellites remain an economical and informative technology for conservation genetics. Ecol Evol.

[11] Hodel RGJ, Segovia-Salcedo MC, Landis JB, Crowl AA, Sun M, Liu X (2016). The report of my death was an exaggeration: a review for researchers using microsatellites in the 21st century. Appl Plant Sci.

[12] Ravaomanana J, Michaud V, Jori F, Andriatsimahavandy A, Roger F, Albina E (2010). First detection of African swine fever virus in *Ornithodoros porcinus* in Madagascar and new insights into tick distribution and taxonomy. Parasit Vectors.

[13] Dupraz M, Toty C, Devillers E, Blanchon T, Elguero E, Vittecoq M (2017). Population structure of the soft tick *Ornithodoros maritimus* and its associated infectious agents within a colony of its seabird host *Larus michahellis*. Int J Parasitol Parasites Wildl.

[14] Pereira De Oliveira R, Hutet E, Lancelot R, Paboeuf F, Duhayon M, Boinas F (2020). Differential vector competence of *Ornithodoros* soft ticks for African swine fever virus: what if it involves more than just crossing organic barriers in ticks?. Parasit Vectors.

[15] Binetruy F, Dupraz M, Buysse M, Duron O (2019). Surface sterilization methods impact measures of internal microbial diversity in ticks. Parasit Vectors.

[16] Lv J, Wu S, Zhang Y, Chen Y, Feng C, Yuan X (2014). Assessment of four DNA fragments (COI, 16S rDNA, ITS2, 12S rDNA) for species identification of the *Ixodida* (*Acari*: *Ixodida*). Parasit Vectors.

[17] Beati L, Keirans JE (2001). Analysis of the systematic relationships among ticks of the genera *Rhipicephalus* and *Boophilus* (*Acari*: *Ixodidae*) based on mitochondrial 12S ribosomal DNA gene sequences and morphological characters. J Parasitol.

[18] Black WC, Piesman J (1994). Phylogeny of hard- and soft-tick taxa (*Acari*: *Ixodida*) based on mitochondrial 16S rDNA sequences. Proc Natl Acad Sci.

[19] De S, Kingan SB, Kitsou C, Portik DM, Foor SD, Frederick JC (2023). A high-quality *Ixodes scapularis* genome advances tick science. Nat Genet.

[20] Jia N, Wang J, Shi W, Du L, Sun Y, Zhan W (2020). Large-scale comparative analyses of tick genomes elucidate their genetic diversity and vector capacities. Cell.

[21] Shah A, Schielzeth H, Albersmeier A, Kalinowski J, Hoffman J (2016). High-throughput sequencing and graph-based cluster analysis facilitate microsatellite development from a highly complex genome. Ecol Evol.

[22] Novák P, Neumann P, Pech J, Steinhaisl J, Macas J (2013). RepeatExplorer: a Galaxy-based web server for genome-wide characterization of eukaryotic repetitive elements from next-generation sequence reads. Bioinformatics.

[23] Castoe TA, Poole AW, de Koning APJ, Jones KL, Tomback DF, Oyler-McCance SJ (2012). Rapid microsatellite identification from Illumina paired-end genomic sequencing in two birds and a snake. PLoS ONE.

[24] Rozen S, Skaletsky H (2000). Primer3 on the WWW for general users and for biologist programmers. Methods Mol Biol Clifton NJ.

[25] Griffiths SM, Fox G, Briggs PJ, Donaldson IJ, Hood S, Richardson P (2016). A Galaxy-based bioinformatics pipeline for optimised, streamlined microsatellite development from Illumina next-generation sequencing data. Conserv Genet Resour.

[26] Schuelke M (2000). An economic method for the fluorescent labeling of PCR fragments. Nat Biotechnol.

[27] Coombs JA, Letcher BH, Nislow KH (2008). create: a software to create input files from diploid genotypic data for 52 genetic software programs. Mol Ecol Resour.

[28] Goudet J. FSTAT, a program to estimate and test gene diversities and fixation indices (version 2.9.3). FSTAT; 2003. https://www2.unil.ch/popgen/softwares/fstat.htm. Accessed 09 July 2024.

[29] Benjamini Y, Yekutieli D (2001). The control of the false discovery rate in multiple testing under dependency. Ann Stat.

[30] R Core Team (2023). R: a language and environment for statistical computing.

[31] Van Oosterhout C, Hutchinson WF, Wills DPM, Shipley P (2004). Micro-checker: software for identifying and correcting genotyping errors in microsatellite data. Mol Ecol Notes.

[32] De Meeûs T, Noûs C (2022). A simple procedure to detect, test for the presence of stuttering, and cure stuttered data with spreadsheet programs. Peer Community J.

[33] Kosman E, Jokela J (2019). Dissimilarity of individual microsatellite profiles under different mutation models: empirical approach. Ecol Evol.

[34] Araya-Anchetta A, Busch JD, Scoles GA, Wagner DM (2015). Thirty years of tick population genetics: a comprehensive review. Infect Genet Evol J Mol Epidemiol Evol Genet Infect Dis.

[35] Cumming D. A Field Study of the Ecology and Behaviour of Warthog. Museum Memoir No 7, National Museums and Monuments of Rhodesia. 1975;7:1–179.

