# Supplementary material for: Development of microsatellite markers for the soft tick Ornithodoros phacochoerus
Source: Parasit Vectors. 2024 Jul 11;17:301. doi: 10.1186/s13071-024-06382-7 (PMC11238500; doi:10.1186/s13071-024-06382-7)
Supplement: Supplementary file 1 — Additional file 1: Dataset 1. Sequencing of Ornithodoros ticks collected in Mozambique for species identification for COI, 12S rRNA gene, and 16S rRNA gene. Sequences assembled using Geneious. [file 13071_2024_6382_MOESM1_ESM.docx]

**Additional information 1: Dataset S1.** Sequencing of *Ornithodoros* ticks collected in Mozambique for species identification for COI, 12S rRNA gene, and 16S rRNA gene. Sequences assembled using Geneious.

> MOZ_Ornithodoros_indiv57.1-COI.ab1

AGTTCCGAATGGTTCCCGCTTTCCTGTTTGAAAACAGATAATATGGGAGATTATTCCAAACCCAGGGAGGATAAGAATATAAACTTCTGGGTGTCCAAAGAACCAAAATAAGTGTTGATATAAAATAGGGTCCCCCCCCCCAGCCGGGTCAAAAAAGGATGAATTAAAATTACAATCTGTAAAAAGTATGGTGATAGCTCCTGCTAAAACAGGTAATGATAAAAGCAATAGGATTGTTGTTACTAGTGTTGATCATAAAAATAAGGGGATTCGTTCAAGAGTTATCCCTTTAGGTCGTATATTTATGATGGTTGTGATGAAGTTAATAGAACCTAAAATGGATGAAATTCCAGCTAAATGTAAACTGAAAATTGCTAAATCAACGGATATCCCAGAGTGGGACATGTTTGAAGCTAAAGGGGGATATACTGTCCAACCAGTTCCTGCCCCATTTTCTACTATAGATGAGGTTAGTAAAAGGCTTAAGGCTGGGGGCAGAAGTCAAAATCTTATGTTATTTATTCGGGGAAAAGCTATATCAGGGGTTCCTAATATAATAGGGATTAGTCAGTTTCCGAAACCTCCGATTATAATTGGTATAACTATGAAGAAAATTATGACAAAAGCATGAGCTGTTACGATTACATTATAAATTTGATCATCTCCAATAAATGAACCTGGCTGTCCAAGTTCAATTCGGATTAAAATTCTTAGTGATATTCCAATTATTATTGATCAAGAACCAAATAATAAGTATATAGTCCCAATGTCTTTATGGTTTGTGGAATAAAATCATCGCGGTAAAAATGGCTG

> MOZ_Ornithodoros_indiv57.1-12S.ab1

AAATTCAAATAAACATTATAATTTCAATTTACTTTTAAATCCTAAACTTATCTTCCAAATCTCCTATATTAATAAAAGTAATTTATTTCAACCTTAAACTTCTGCTGCATTTTGACCTAACATCCTAACTAAATATAATCTTAATTATTAGTTAATCAACATCATTCTATGATAGCGATATACAAGCTGCTTAACCAATTTAAGTAAGATTCTTGAGTCTTATCCATCAAAGAATAAATTCCTCTAAAAAGCTTAAAATACCGCCATAATCTTAAGATTTAAAAAATCAAAAAATACTACCAATCTTAAATTTTCGAATAACAGGGTATC

> MOZ_Ornithodoros_indiv63.1-12S.ab1

CGATGTGTGCATATTTTAGAGCTAAATTCAAATAAACATTATAATTTCAATTTACTTTTAAATCCTAAACTTATCTTCCAAATCTCCTATATTAATAAAAGTAATTTATTTCAACCTTAAACTTCTGCTGCATTTTGACCTAACATCCTAACTAAATATAATCTTAATTATTAGTTAATCAACATCATTCTATGATAGCGATATACAAGCTGCTTAACCAATTTAAGTAAGATTCTTGAGTCTTATCCATCAAAGAATAAATTCCTCTAAAAAGCTTAAAATACCGCCATAATCTTAAGATTTAAAAAATCAAAAAATACTACCAATCTTAAATTTTCGAATAACAGGGTATCTAA

>MOZ _Ornithodoros_ indiv24.3-16S.ab1

TTAATAAAGGTATCCTAATCCAACATCGAGGTCGCAATCTACTTTATCTATATGAACTATCCAAAGTAATAACGCTGTTATCCCTAGAGTATTTTTTTTAAAATATCAATAATATTGGATCCAATAATACATTTTTAAAGTTTTTAAAAATTTAAAAATCGCCCCAATTAATTAAAAACTTTCCTTGAATTTTTTTAAAGGACCAAAAAAGAAAAGCCTTTAAAAAAATTCATAGGGTCTTCTTGTCCCAAAAATTCATAAAAACTTTCTCATTTTTAAATTAACTTTTAATATAAGCAAAAAAAAAGCTTATTTTTGTTAAACCATTCTCTTAGCACCCAATTAAAGTCTTATTTCAATACCTTCGTATAGTCATAATACCACAGCAATTTAAAAAAT

>MOZ _Ornithodoros_ indiv69.10-16S.ab1

CTTCTTCATTAATAAAGGTATCCTAATCCAACATCGAGGTCGCAATCTACTTTATCTATATGAACTATCCAAAGTAATAACGCTGTTATCCCTAGAGTATTTTTTTTAAAATATCAATAATATTGGATCCAATAATACATTTTTAAAGTTTTTAAAAATTTAAAAATCGCCCCAATTAATTAAAAACTTTCCTTGAATTTTTTTAAAGGACCAAAAAAGAAAAGCCTTTAAAAAAATTCATAGGGTCTTCTTGTCCCAAAAATTCATAAAAACTTTCTCATTTTTAAATTAACTTTTAATATAAGCAAAAAAAAAGCTTATTTTTGTTAAACCATTCTCTTAGCACCCAATTAAAGTCTTATTTCAATACCTTCGTATAGTCATAATACCACAGCAATTT

>MOZ _Ornithodoros_ indiv71.9-16S.ab1

CAATCTACTTTATCTATATGAACTATCCAAAGTAATAACGCTGTTATCCCTAGAGTATTTTTTTTAAAATATCAATAATATTGGATCCAATAATACATTTTTAAAGTTTTTAAAAATTTAAAAATCGCCCCAATTAATTAAAAACTTTCCTTGAATTTTTTTAAAGGACCAAAAAAGAAAAGCCTTTAAAAAAATTCATAGGGTCTTCTTGTCCCAAAAATTCATAAAAACTTTCTCATTTTTAAATTAACTTTTAATATAAGCAAAAAAAAAGCTTATTTTTGTTAAACCATTCTCTTAGCACCCAATTAAAGTCTTATTTCAATACCTTCGTATAGTCATAATACCACAGCAATTTAA

>MOZ _Ornithodoros_ indiv72.3-16S.ab1

TTAATAAAGGTATCCTAATCCAACATCGAGGTCGCAATCTACTTTATCTATATGAACTATCCAAAGTAATAACGCTGTTATCCCTAGAGTATTTTTTTTAAAATATCAATAATATTGGATCCAATAATACATTTTTAAAGTTTTTAAAAATTTAAAAATCGCCCCAATTAATTAAAAACTTTCCTTGAATTTTTTTAAAGGACCAAAAAAGAAAAGCCTTTAAAAAAATTCATAGGGTCTTCTTGTCCCAAAAATTCATAAAAACTTTCTCATTTTTAAATTAACTTTTAATATAAGCAAAAAAAAAGCTTATTTTTGTTAAACCATTCTCTTAGCACCCAATTAAAGTCTTATTTCAATACCTTCGTATAGTCATAATACCACAGCAATTTAAAAAA
