# Supplementary material for: Development of microsatellite markers for the soft tick Ornithodoros phacochoerus
Source: Parasit Vectors. 2024 Jul 11;17:301. doi: 10.1186/s13071-024-06382-7 (PMC11238500; doi:10.1186/s13071-024-06382-7)
Supplement: Supplementary file 2 — Additional file 2: Dataset 2. Sequences of the twenty-four microsatellite markers designed. [file 13071_2024_6382_MOESM2_ESM.docx]

**Additional information 2: Dataset S2.** Sequences of the twenty-four microsatellite markers designed. In the name of the sequence is indicated the microsatellite number (MSXX), the name of the genomic dataset and information from the genomic dataset (Forth, J, L Forth, S Lycett, L Bell-Sakyi, G Keil, Blome S, Calvignac-Spencer S, et al. 2020. “Identification of African Swine Fever Virus-like Elements in the Soft Tick Genome Provides Insights into the Virus’ Evolution.” BMC Biology 18 (1). https://doi.org/10.1186/s12915-020-00865-6.)

>MS2_OporcMADA_NODE_61806_length_939_cov_2.443350

GTCGACAATTTCTCTCGCCCTACGAAAAAAATACTAATAAGAAGTAAACCTCACTTCTACGTCGCCTTCTTTTACGTTTTACAAATCGAACATCACATCCCAGGAATTTATCCAGGGAGGTTCCTTAAAAGCACACACACACACACACACAAAAAAGACTAAAGGTATCGGAGGCTGCATCCTCTTTTCGACAACCCAAACGGGAAATAAATTTGTCCGCCAGCGGCTGTTTGGAAGCAGCATTTCATGAAAAGGGTCGGTATGGAGGAAGGGAGACCCATTGTTTGGGAA

>MS24_OporcKENYA_NODE_18175_length_989_cov_2.276102

TGTTTACGACGGCATGAAGCGGCGATCTCGTTCGCTGTAAGGAGTGGCCACGTACGACGGAAGGGCCTGGTCGCAGCGCTTGCTGTGCGTGCGTCCGTGTGTATGTGTGTGTGCATACACGCAAGTGTAGTTGAGGTTGGAAAAGGGAATTTCATTGTGTGGGTGATTCTATGGAGTATCTGCTGTTTATGGATTTATGGCGTCAAGAATCACGTTGCTGTGCTTTCTGCTAACGATAACATCGTCGTACAGCTGCAGTAAAGGTGAGCAACGTTCGAGCTTTCGTATTTTCCGC

>MS30_OporcMADA_NODE_28604_length_1905_cov_2.782902

AGGGTGCCCTCAATACAACGGCTTCGGAACGTTATTCTCTTTCTGTGCATGTTTGCTTGCCTTCTGGCCGACAACAGAAAATGTTTCAAGCGGAACCGTTTTGAAAAGGTCGCGCAGCCAGAGCTTTTTAGTGAAGTCATATACAGAACAGATATGAAGTATATATATGTATATGTGTGTGTGTGTGTGTACACCGTGCTGTCGCTGCTTACATCATGCGCACACA

>MS35_OporcMADA_NODE_43663_length_1393_cov_2.638231

CTCAGGTGTCACCAGCAAGCTGTGTATCTGTTCATATATATATATATATATACACTATAGGAGCCTAGACATTGTCGGG

>MS46_OporcMADA_NODE_34879_length_1666_cov_3.922677

TAGCGTGAACATAGCGGTGGAAACAGCACACAGATTCACACACGATTTGGTTGAGAACGTGCGTTAAAGCCGCAGATTAAAACTGTGTTAAAATCAGTAAACAGTGTAGTATAGCATACCAAATGGTCTTCATCTGATACTGCTTAACCTTGGTGGTGCATGATGTATTATACCAGCATTTCGAAACACTGGATAAAAAACACACACACGCACATGAAATTGGGACGAATGCACGAAGAAATGCCGTCCACGTCATTACATGGGCAACCAACAACCTTTACACAACGGCAAGAAATTCCAATATTCGCTCCTCTTTTGCTTTCAACTGCAGTCTTCCCTCACGGCACGTGCTTCGCATTCCTTCCGGGAAAACTTCTCC

>MS48_OporcMADA_ NODE_553_length_8605_cov_3.955178

TCTGCTTTTCAAGGCTGTGCACTGCATTGAAAAGTAAGACCTTGGCACTATCTATCATCAGTTGAACTCACAAGATACACACACACACATTATTATGTAGCTATTACTCTTTAGCCATATTATTGGGCACCCCTAACGGATAAGTAATCACAAGCACAAACACCACACGAATGACCATGGGGAGAAACAAACTGTGACTGACGTGTCTAAAACTTCAGCACGTTTTGCAAGGTAACAGGCTCCGAA

>MS59_OporcMADA_ NODE_31808_length_1778_cov_2.090248

ATAGAGGCAAGATGGCAGGCTAACGGACCGTGTTACGGTTTACCATTTGCCCGTTTCTTTTTTGTTTTGTGTGTGTGTGTGCGCGCGCGCATGTGTACACATCTTCAGACTTTATGAAGTATGAATTTATGATAAGGAAGGGGAGGGTGCCATGTTGCATAGGTCAACCATTCTAGCTCCCTAACTTCTGATACAAGGGAAAGGCTCATTGCATCACGAACTGGAGATTGCAGACTACCACGTCGGGGGCTGTACTTCCCTTTCACCACTAGTTTATCTGTTACAAGTCTCTTGCTCTGATATACTTTTTGTTTGTGCGTCTTCCCCTTACCCAAGCTCCCCTGTCTCCAAGCAAGGAAGAATTCGTCCACCCGTTTCAGCACCATGCTACCTTGAACTTGCACAGCTGG

>MS61_OporcMADA_ NODE_6403_length_3963_cov_3.938999

CAGCGAAACAAGCAATGAGCTCATCCTGTTCTTCTGTCAAACGGGGATGACCTTTGCCATTCATGTAATCCTGTTTCGTTTTTCGCCGTACCCGAGTGCCATAATGTGGCCGCATATATTCAGCAACGCCAATTATTATTATTATTATTATTGATTTGAGCATTCATGTTATCTGAATTTTGCTACATTAGAAATGAGGCATTCTGACATATTCTTTTCATAAGATTGATGCATATTTTGACGGTCATTTGTAATGAGTTTGTTGCAACTGAAAATTTGTTAAGAGTGGGTCGTTGTAACCGGGATTTGCT

>MS63_OporcMADA_ NODE_4141_length_4665_cov_2.753195

CATGCTCACAGTGCTTGACGTCATCTTTAACCGTTGCGTAATAACGTAATTAGGGTGCCGTTTTTATAGGAGACAGGCGCACGTGGAAGTGGTTCGCCAACACTAGACCAAAATCTCCCTCAATATTTACTACGTTCTTGCTTATGTGTGTGTGTGTGTGGGAATACGAACTCAGTGCCTCTCCGCTCCCCGAATTCATCCTCCATGAAATATGATAAGCGCGCAGACCAAGGTAAATTATCTTATCTCGGTTTAAAAGAGCATACTGCGGCGGAAGCGTCACGGAGAAGAGCAGATTGGGAAAGGAAGAGGGACTGCAGCTAAATGACATGCCTCCGGTGCACCTGGCCACTGCAAGGCCTATTTCATTGGCCACTATGTCATGATGTGGTCCCCTCTGGTCATGTGACAA

>MS64_OporcMADA_NODE_27976_length_1931_cov_2.512195

CGGACAGAAATAGCGGAACCCTAGATCGTATTACAAGACTATTCAGCAGACAGATCAAAACGGCACTAGATTAGAGAATGACGTTACTAGCACCGTGACAGGCAATTATACAGCGTGATACGTGCACATATCAGGCTGTTGCTCTTGTGTTGTGGCTGTAACTGCATTGCTCTACCCTCTACCTGTGTAGACAAACAAGTGTGGAAAATTGTACATATTGTTACCGTTACCGCAGTTGCTTGCTACGCGTTACGTTTTCCACCCACCCCGCTTATATATATATATGTATATATATAAAGAAACAAAGTAAGAAATACTCAATACATAAATAAGTTCAGTCATTGAACCAGGGTTCAGGCTACGCATTTTCTGGTCTTTGGTGCTTCTTTGCTTGATTTATTTTAGTATTTCTCGTCTTACAGATGAAGAAACTGCGCACACTGCATAGGCGCATCCCTGCGTTTGGTTAT

>MS66_OporcMADA_NODE_1718_length_6233_cov_3.385195

CTTCCTTCTGATTGAGCGGCCGTGCCCGTGGGTATTGGTAGATTCGAAACGGACTTCTCCCTTTTCGCAGAAGACGAAGAAGAAACCTCATCCTCTTCCTCCATCGTCGTCTTCTTCTTCGTCATCCTCCTGTTACAGGATCACACAGAAGAAGAAGAAGAAGGATCCCCTCCACTCTTGCATGTATTTTCTTGTTCGGATTGACATATTTAGGACATTGAGATTAAGATATCAAAGGCTCTTTCCAGCATTCATACATCAACAGCCTATACTACTTCTCTCCACATTAACTGTAAGTGGACGCCAAACGCCCTGGCAAATTGCCGGAGAGGAGGAGAAGGAAGAAGAAAGGCCACCGTTTGTGTCTTCAA

>MS71_OporcMADA_NODE_15240_length_2710_cov_2.805265

TTCAGATTCACAACAGGGCGGGTGGAACAGTCCTACCATTTCTGTGAAAGATGATGATGATGACGACGACGACGAAACTGAGGAAGAAGAAGAAGAGGAGGAAGATGAGGACAGTGACCTTATGTGCACTGTATGCAGCAAAGGGGAATCTGAGCCTCCCAACGAGATCGTCATATGCGACACATGTAATAAGGGTGAGAGCACGTTGAATGC

>MS73_OporcMADA_NODE_24090_length_2114_cov_2.711122

TTCGGATTCGAACAAACACGTGAAGAGAGAGAGAGAGAGAGAGAGAGAGAGAGAGAGAGAGAGAGAGAGAGAAAGGCGTTTCATTCATCCATATATCACTGCGATATTCTCGGTTTCGATCGCCCACACAGGATCGATTCGCTGCATGCGTTGTATAATGTTTACATGCGCTGAAAAAGAAAATGTGTATATAGGATGCAATAATGGCCATTACATATTTTATTCACTTTCAAATCTACCATTTTCTTTTCCTCCGTTGATGGGTTTCAGTATCCGTACGAGTTGAGTTTCAATGTTTGATGACGTACTCTCTCCCCACATGGGTGTCACGGAGAAGCAATTGCGTTCTCTTTTTCTCCTTTCTTTCTTTTTCCACGAAACAAGGCTCGGGCATTGGAGCGTCGGAGTCATTCGTTCTATAATATGCAGAGATGATGGAAGTGAGAGGGCACGAAC

>MS76_OporcMADA_ NODE_106842_length_566_cov_3.111617

TCTTACGCTGAACATTGGCGAAGCGGTGAATGTTTCTTATCTGATTTAGAAAGTTACCGCTGTGTTAGAGAGAGAGAGAGAGAGAGAGAGAGCAAAAGAAAAAAAAAGGAGAGAAAGAGAACAATAAGTGAAGAAGGAAAATAAACAAAAACAAGAAAGATACACAAAGGCTCTCTCTGTCGCTTCGCTTCCTCCCAGTGCAGTAGCAATT

>MS78_OporcMADA_NODE_106874_length_566_cov_2.548975

CTATCACGACGCCTCCTTCCTTTCCCTTCTATTCTCTTTTTTTTTTTGTGTGTGTGTGTGTGTGTGTGTGCTCTGTAGGGGTTGGTAAAGTTCAGTGTGGAACGTTGAGGTGTTTCTGCTGGGTTACGACGAGGCCAAATTACGCCGGCGTATCTAGTAACGCAGTTGTGGATGTTACGCATTGACGGTTCGCTTGCCTTGAGTGAATTGGTGGGACGTGACGAAAACAAAATGGCTGTGAACGTGCCCGGAAAGGTTTTGTTTCTCTTGCCCGTCATTGCTGAGCTTCAG

>MS81_OporcMADA_NODE_22817_length_2182_cov_2.570316

CCCTTTGACAAACCGTAGGCACATAGAACCCGTCACAATGGCCAGTGATATTTTTTCGTCATCTAAGCTCTTTGTGTTGGTGCGCAATGACCGCGCCTATGTGTGACCATTGTCCAGAAGGAAGCATTTACATGCGAAGCTTTCGGACGAGAAAGATGTCAGCAGAGAAAACAACCCTGATGCAGTTTAAAGTGCACCGCTTGTGCTGAAAAAGGCACAACGAAAGGATGACTAACTGACACCTTCTCTACCTCGATCGCTTGGCAAGCTCATCTTATGTCCTGCCCTTCTGCTTGTTCCTTTGTTTTCTCTCTCTCTCTCTCTCTGTTTGATATACGGGGTGATCACTTTTAATCGATAGATTTTTTTGCGATACAGGATATGTTGCGATAGCGCTACTCGCACGCTGTCTGCGCCGATGAGAAATACGGTCTGGCGAAAATGATTT

>MS82_OporcMADA_ NODE_46251_length_1303_cov_1.549320

CAGTTCAGTTTACGCTCGGCGTCGCACAGTGTCTGCCATATATATATATATATATAAAATCCCCTCAAATTTATGAGAATGTGCCCCGCCTGTTTCACGGTATTTTTCACAATAAAGTTTGTAGCGTAATCTGCGTACTCTGTCACTGCTCTTCGTTGATCACTTCATTTCGAAAACAGGTGTAGAGGATATCAAATTTTGAATGAAAACATGAAAACATATTTAGGAGGTAACGAAGCTCAGTTGATAGCGAGTGTAACGGCCTTCAAAACATTATGCTTTCTTTTACGTGCGGTCATTAATACGCTCATAGTTCCTTTTTACTTTCGTCCTCGCACAAAAGGAGCTTTGAAGGTACCGCATAAACGGGAGTAACATAACAGGAAGATGGGTTCGTTTGGTAACGCAAGCTCGGAACCTGGTGGAGGATACAAATGCTCGCGCGGGGAAGAATATTTGTCCGAACCCAATTCATGGAGT

>MS87_OporcMADA_ NODE_64413_length_901_cov_1.851421

ATGAAGCGATCGTCCTACGGCTTCAAGAATCTGCTAAACGGAGAAAAAACTACGAAAAAAAAAAAAAAAAAGAAGATGAACAAAACGCGGGAGGTGTTCCTAACTATATCCTTTAACGCGAAAGTGTACAGCTTTGGTGATGCAGGGCAAGTTAAAACGCTGCATGTGAAGTTAATGAAAGTAGTTTGAGCATATTCAGAGATGGGTAGGTTGTCCCAGAAAGTGTCCTGACTTTTTAGCGTCTCATGTCCCGGTTCCAGCTTCTCTCTCTCTCTCTCTCTTTTTTTTTTTTTTTGTGTGTGTGTTTGCATGCCGGCAGTTCAAGATCTACATGCCCTGAGTCTGGGGGAAAACTGGACGAAGACAAACAAATAAGGAGTCTGCAAAAACAGAAAATGACGCTTGTATGAAAACGGCAATGAACACAACAACGAGGTTATACGAAATGAAGAGACGAGGCGAATCAGGAAAGCGTCTC

>MS90_OporcMADA_NODE_633_length_8321_cov_3.025384

TGAATAACGGGGTAAAGCCGGAAGCCGAAGAGGGTGCGAAGTCCGTCCGAACCACAGCAACGCCAAATCCATAAATGCGGGCAGGGACGGCACGAGACGGAAACGTGCAGGATCGTCCCGTGCAGCACAAGCCCTGCGAGGCACCCCCACAGCACTCCGGTAATTGGAAAAGGGGGTGAGGAGTGGAAGAGAGAAATTTTCATTAACGGCGCGGCAAGGGGAGCTGCTGCTTCCATGATAAACATGCAGCCGAGAACGCGTTGCTGCGCACGCGATGCATTATACCATCACAGCCAGTTAAAAGGAGGGAGAGGAGGAAAACAGAGAGAGAGAGAGAGAGCAGCAGCAGGCGGTGTAAAATCTGAACTCCGTGCAGCAAAGGACAGAGCAGGACTTCGTTGTGTTTTTAAGTTTGCTGCCAAACAGCTATTTGCTAAGCACGAATACAGCACTCCCA

>MS96_OporcMADA_ NODE_4256_length_4614_cov_3.217517

CCACCCCTCTAGAACCCTCCGGTTGTGGTTGAAGTGCTTGAATAATTGTATCAACTGGGGGAAAAAAGATGTAAGCAACTGAGGGTACCGTTTTCTTTTTTACTGCTCATTTGTTCCTGGAAATGACACTAAAATGAACGGCTGCCTTTTTATTTCTATTGCTCGCGTGGGATACATATTGTGGACTTTACGCCTAGAATCACGGCGGCGCTTTCAGAAAGAGTTGCGCGGCGGAAGTTCTCAGTAATCGTTTCCTTTTTTCCTTTGTGTGTGTGTGTGTGTCCATGTGTTTCCTATAGGCCATCTAAATAGGAAGGGGTTGCCGTTCAGCCAGCTTAGAT

>MS101_OporcMADA_NODE_15902_length_2652_cov_2.466931

GGCTCACGAAAATACCTCGCAGCCTTGGTAATTATATGGACTCTTCAGACGAAACAAATAGGAGTACGTAGAATGCACTCGAATTCACTGGGGCACAAAATAAACAGAGAGAGAGAGAGAGAAAAAAAAAAAGCCTGAAGGGAGCATACCGTTAGCTGG

>MS102_OporcMADA_NODE_12915_length_2934_cov_2.863555

TGCGCCTACTGTGTACCACCACTGATGAGACTAGTCTCTTTCTTCTTTTTCCCCGAAACTTCGCTTTTTTTTACTCTCTCTCTCTCTCTCATTCCCTGTCCTAGCATTTCTTAGTTACTGCATGTTCTACCCGTTTACTATAGAACCATGTTGGTGATACGTGGGTTTCAAAGAACACTATAGTGAGTTGGAATAACTAACCATCAGGAGAATCTGCACACGAGGAACTGATCGTGAGGTGTTGCTGTGTGGTTATCTGAAGCTTGCGGG

>MS111_OporcMADA_ NODE_13086_length_2917_cov_2.724014

CCAAAACACTGGATGAAGCCAGGAGGAGGAGGAGGAGGAGGAGGAAAAACAAACAATAAGAAATGTTTCCTCTGTCTGAGGAACTTGAAGTTGCAATTTTCTTGTGGTTCCTACGGTTGAGCGAC

>MS117_OporcMADA_ NODE_13944_length_2829_cov_2.491858

CGCACTCATTGAGAGTTCGCCTGCAGAAGGAACCTGTGCACTTAGCGCAGATAAAAGTGCTACTAGGCTGCTTTTATCGTGCAGCAAGCAGTAACTTCGCGGGGTGACCGAGATAACAGTGATTCGATGACCAGCCTGCTATCTCAGCGAAAGTGGGGAGCAGCACAGTCGTACTTCCTGTGTCGCGTTATGTGCTGCAGAACCTTTAGGTTTGGATAGCAGGACAGTACGTACCCCCGTAACATCTGTTTGCATTTTTCGCGTAACATGTAGAACATACTAATAAACGTATACATAAAAAATAGCCCACAGAACCGCTAATCTGTATGAGAAGCTTGCTGCGCATCTTTCTTTTTTTTCTTCTTTTTTTTTTTGTGTGTGTGTGTGTGTTAATCGACTACACTCACAGGTGGTGCCAGCCATCACGGAAACGTTAAA
