# Supplementary material for: Development of microsatellite markers for the soft tick Ornithodoros phacochoerus
Source: Parasit Vectors. 2024 Jul 11;17:301. doi: 10.1186/s13071-024-06382-7 (PMC11238500; doi:10.1186/s13071-024-06382-7)
Supplement: Supplementary file 3 — Additional file 3: Table S1. Repartition of twenty-four microsatellite markers in three multiplexes for amplification and genotyping on O. phacochoerus samples. For each genotyping multiplex (named Plex1 to Plex3), two PCR were performed separately (PCR1 and PCR2) with four loci (Column Locus name) in each PCR marked with four different fluorochrome (Column Fluorescent dye). According to preliminary tests, PCR products were diluted (1:dilution) before being pooled together to form the genotyping multiplex. For each locus, the expected sequence length based on O. porcinus genomic data is indicated as it was taken in consideration for the repartition of fluorescent dyes between the loci (two loci with the same dye in the same genotyping multiplex needed to have different lengths to be distinguished in the final electropherogram). Finally, all fluorescent dyes did not lead to the same levels of amplification during PCR, consequently, primer concentrations were adjusted for each locus (Primer concentration) in each PCR to obtain similar level of fluorescence in the end. 1 The sequence size for locus ms-76 in O. phacochoerus ended up being longer (386bp) than the expected size. [file 13071_2024_6382_MOESM3_ESM.docx]

**Additional information 3: Table S1.** Repartition of twenty-four microsatellite markers in three multiplexes for amplification and genotyping on *Ornithodoros phacochoerus* samples

| **Genotyping multiplex number** | **PCR  multiplex (1:dilution)** | **Locus  name** | **Fluorescent dye** | **Locus  length (bp)** | **Primer concentration** |
| --- | --- | --- | --- | --- | --- |
| Plex1 | PCR1 (1:1) | ms-76 | FAM | 211^1^ | 0.1µM |
|  |  | ms-35 | VIC | 79 | 0.15µM |
|  |  | ms-48 | NED | 246 | 0.15µM |
|  |  | ms-78 | PET | 291 | 0.2µM |
|  | PCR2 (1:1) | ms-59 | FAM | 410 | 0.1µM |
|  |  | ms-96 | VIC | 341 | 0.1µM |
|  |  | ms-81 | NED | 448 | 0.15µM |
|  |  | ms-64 | PET | 470 | 0.25µM |
| Plex2 | PCR3 (1:2) | ms-71 | FAM | 213 | 0.1µM |
|  |  | ms-111 | VIC | 125 | 0.15µM |
|  |  | ms-102 | NED | 270 | 0.15µM |
|  |  | ms-24 | PET | 295 | 0.2µM |
|  | PCR4 (1:2) | ms-63 | FAM | 412 | 0.1µM |
|  |  | ms-66 | VIC | 371 | 0.15µM |
|  |  | ms-73 | NED | 456 | 0.15µM |
|  |  | ms-87 | PET | 478 | 0.2µM |
| Plex3 | PCR5 (1:10) | ms-30 | FAM | 226 | 0.1µM |
|  |  | ms-101 | VIC | 159 | 0.075µM |
|  |  | ms-2 | NED | 286 | 0.225µM |
|  |  | ms-61 | PET | 311 | 0.2µM |
|  | PCR6 (1:1) | ms-117 | FAM | 438 | 0.1µM |
|  |  | ms-46 | VIC | 379 | 0.15µM |
|  |  | ms-90 | NED | 457 | 0.125µM |
|  |  | ms-82 | PET | 480 | 0.225µM |

For each genotyping multiplex (named Plex1 to Plex3), two PCR were performed separately (PCR1 and PCR2) with four loci (Column Locus name) in each PCR marked with four different fluorochrome (Column Fluorescent dye). According to preliminary tests, PCR products were diluted (1:dilution) before being pooled together to form the genotyping multiplex. For each locus, the expected sequence length based on *O. porcinus* genomic data is indicated as it was taken in consideration for the repartition of fluorescent dyes between the loci (two loci with the same dye in the same genotyping multiplex needed to have different lengths to be distinguished in the final electropherogram). Finally, all fluorescent dyes did not lead to the same levels of amplification during PCR, consequently, primer concentrations were adjusted for each locus (Primer concentration) in each PCR to obtain similar level of fluorescence in the end. ^1^ The sequence size for locus ms-76 in *Ornithodoros phacochoerus* ended up being longer (386bp) than the expected size.
